# What Behavioral Abilities Emerged at Key Milestones in Human Brain Evolution? 13 Hypotheses on the 600-Million-Year Phylogenetic History of Human Intelligence

**DOI:** 10.3389/fpsyg.2021.685853

**Published:** 2021-07-29

**Authors:** Max S. Bennett

**Affiliations:** Independent Researcher, New York, NY, United States

**Keywords:** evolutionary neuroscience, evolutionary psychology, evolution of theory of mind, evolution of episodic memory, primate intelligence

## Abstract

This paper presents 13 hypotheses regarding the specific behavioral abilities that emerged at key milestones during the 600-million-year phylogenetic history from early bilaterians to extant humans. The behavioral, intellectual, and cognitive faculties of humans are complex and varied: we have abilities as diverse as map-based navigation, theory of mind, counterfactual learning, episodic memory, and language. But these faculties, which emerge from the complex human brain, are likely to have evolved from simpler prototypes in the simpler brains of our ancestors. Understanding the order in which behavioral abilities evolved can shed light on how and why our brains evolved. To propose these hypotheses, I review the available data from comparative psychology and evolutionary neuroscience.

## Introduction

Humans have an incredibly diverse suite of intellectual faculties. We can build cognitive maps, infer intentions of others, remember specific historical events, communicate with each other using language, learn motor skills through observation, and more. But all these varied faculties, which emerge from the complex human brain, are likely to have evolved from simpler prototypes in the inevitably simpler brains of our ancestors. This general idea of progressive complexification of behavior from simpler roots has been elegantly articulated in Paul Cisek’s theory of “phylogenetic refinement,” whereby an extant animal’s behavioral repertoire is interpreted as a consequence of evolutionary refinement from more basic phylogenetic building blocks (Cisek, 2019). A challenge to interpreting human behavioral, intellectual, and cognitive faculties through the lens of phylogenetic refinement is in identifying the faculties present in our ancestors, as these were the building blocks upon which the process of phylogenetic refinement operated.

To aid this tracking of the phylogenetic refinement of behavior, this paper presents 13 hypotheses regarding the specific behavioral abilities that emerged at key milestones during the 600-million-year phylogenetic history from early bilaterians to extant humans. Given the breadth of this topic, the scope of this paper is narrowed in three ways. Firstly, it develops hypotheses only on phylogenetic history. Secondly, it develops hypotheses only on the *human lineage* from early bilaterians to extant homo sapiens. Thirdly, it develops hypotheses regarding the subset of behaviors that are frequently considered as “intelligent.” I will briefly review each of these three refinements to clarify the scope of the analysis herein.

### Focus on Phylogenetic History

Tinbergen’s Four Questions (Tinbergen, 1963), provide a useful tool for categorizing the levels at which behavior can be explained. His four questions are:

(1) Phylogeny: what were the evolutionary *steps* by which this behavior came to be? What function *did* the behavior serve in the environment in which the behavior originally emerged?
(2) Function: what was the *current* function that the behavior serves, as measured by reproductive and survival success? Note that the original function of a behavior is not necessarily the same as its current function.
(3) Mechanism: what are the underlying (neural, hormonal, biomechanical, etc.) mechanisms by which this behavior is implemented?
(4) Ontogeny: how does this behavior emerge in the development of an individual organism?

The scope of this paper is to investigate the phylogenetic history of behavioral abilities, and as such sets its focus on only one of Tinbergen’s questions: phylogeny. Of course, these questions not entirely separable; mechanisms, functions, and ontogeny provide essential clues to the phylogenetic origins of behaviors. As such, mechanism, function, and ontogeny will be invoked as evidence in favor or against various speculations regarding the phylogenetics. However, the hypotheses themselves stake claims only on the phylogenetic history of a behavior, not its mechanism, present function, or ontogeny.

### Focus on the Human Lineage

The scope of this paper is intentionally anthropocentric – it seeks to chronicle the phylogenetic history of behavioral abilities in the *human* lineage from early bilaterians and extant *Homo sapiens*. This requires an essential caveat to the hypotheses presented here. Proposing a hypothesis regarding the emergence of abilities along the evolutionary lineage from early bilaterians to humans is not the same thing as proposing a hypothesis regarding a unique ability of humans relative to other extant animals alive today. For example, the hypothesis that episodic memory emerged in early mammals is not the same as a hypothesis that *only mammals* exhibit episodic memory. Convergent evolution is not the exception, but the rule. Flying evolved independently multiple times (Ben-Hamo et al., 2016). Lens-based eyes evolved independently multiple times (Ogura, 2004). Alas, as we will see, the evidence is quite strong that episodic memory also evolved independently numerous times – amongst cephalopods, birds, as well as in mammals. Further, extant animals today independently evolved abilities that have never been present in the human lineage (such as the electroreception of certain fish and echolocation of bats). As such, the hypotheses in this paper should not be used to make comparisons between *Homo sapiens* and other extant species.

### Focus on “Behavioral Abilities”

The scope of this paper will attempt to focus on the phylogenetic history of what I will call “behavioral abilities.” I define a “behavioral ability” as an intellectual or cognitive faculty that animals are capable of invoking. I use this term for two reasons. Firstly, I use “behavioral ability” instead of “behavior” because the scope is not intended to review the entire behavioral repertoire of our ancestors. Further, the term “ability” is meant to focus the analysis to the realm of purported animal intelligence – attempting to understand the capacities afforded by the brains of our ancestors. Secondly, I use the term “behavioral ability” instead of “cognitive capacity” or “intellectual capacity” to avoid being restrained by pre-existing definitions of cognition or intelligence, and instead remain agnostic as to the differences in the many purported definitions of each. I use this novel term “behavioral ability” while acknowledging this refinement of scope will be imperfect. What gets categorized as a faculty deemed intelligent is ripe with an unavoidable terminological quagmire and anthropocentrism. Despite this challenge, the chronicling of such behaviors is still fruitful and illuminating as to the function and mechanisms of brains.

### The Logic of Hypothesizing the Emergence of a Behavioral Ability

**FIGURE 1:**
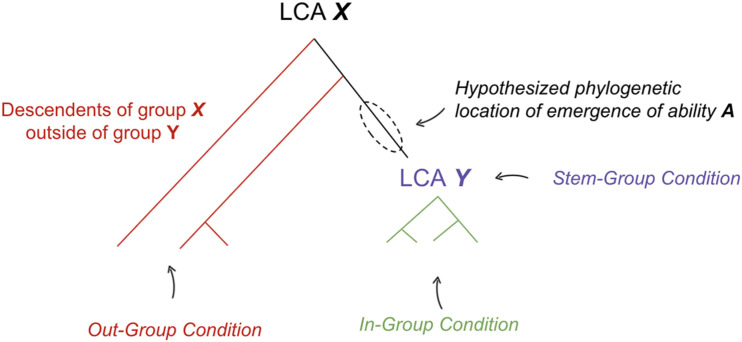
Method for hypothesizing the phylogenetic location where a behavioral ability emerged. LCA, last common ancestor. See text for definitions of each condition.

The hypotheses presented here all take the following form: “behavioral ability ***A*** emerged at some point amongst the early stem members of a phylogenetic group ***Y***” Figure 1.

To support such a claim, evidence is presented in the form of three conditions:

(1) In-Group Condition: Diverse groups of early diverging species across group ***Y*** contain ability ***A***, implemented in homologous neural mechanisms with shared ontogeny.
(2) Out-Group Condition: Evidence is supportive of one of the following three claims: (a) descendants of earlier diverging phylogenetic group ***X*** outside of group ***Y*** do not contain ability ***A*** or (b) ability ***A*** is implemented in non-homologous neural mechanisms with different ontogeny in earlier diverging group ***X*** outside of ***Y*,** relative to species within ***Y*** or (c) principle of parsimony suggests a homologous neural substrate was repurposed for new ability ***A*** within early members of group ***Y***. The principle of parsimony states that if evidence is equally supportive of multiple phylogenetic relationships, we prefer the evolutionary tree that requires the fewest number of evolutionary changes (Fitch, 1971).
(3) Stem-Group Condition: Ability ***A*** would have been adaptive within the purported ecological niche of early members of group ***Y*** (i.e., the now extinct “stem-group” of ***Y***, especially those likely to be the last common ancestor of group ***Y***).

If the preponderance of evidence is supportive of the in-group, out-group, and stem-group conditions, then this is considered meaningful evidence in support of the hypothesis that behavioral ability ***A*** emerged at some point amongst the early members of a phylogenetic group ***Y*** and was thereby inherited by many of its descendants.

The relevance of criteria #2c is subtle but important. There are cases where shared neural structures are *independently* repurposed for similar functions. A simple example of this is that of wings. Both birds and bats repurposed the same structure (front legs) for a similar ability (flying). Although bird and bat wings share homology, the last common ancestor of birds and bats did not have wings. It is reasonable to see why this happens: given the constraints of evolution, different species that find themselves in ecological niches with similar selection pressures may end up similarly repurposing older structures for the same function. In such cases, the true phylogenetic origin of a behavioral ability can only be deciphered either through closer look at the actual substrates (identifying revealing differences in bird and bat wings) or through the principle of parsimony. Most non-mammal amniotes do not have wings, hence by principle of parsimony one would argue that it is more likely that wings were independently gained in the bird and bat lineage, as opposed to being independently lost across all other non-mammal amniotes (which would have required comparably more evolutionary changes). In the case of wings, fossil records are a useful adjudicator, but when it comes to brain structures, fossils are much less informative and hence we must rely on these other strategies to deduce the neural structures and the behaviors they enabled.

I am intentionally broad with respect to the specific evolutionary timing, focusing on the major divergences (e.g., comparing early members of Vertebrata with early members of Mammalia), and am less specific on more detailed timing (e.g., comparing early members of Mammalia with early members of Placentalia or Boreoeutheria). With the foundation of these broad hypotheses, however, further work can add additional detail to the steps of phylogenetic refinement by which these abilities emerged.

## Behavioral Abilities That Emerged in Early Bilaterians

**FIGURE 2:**
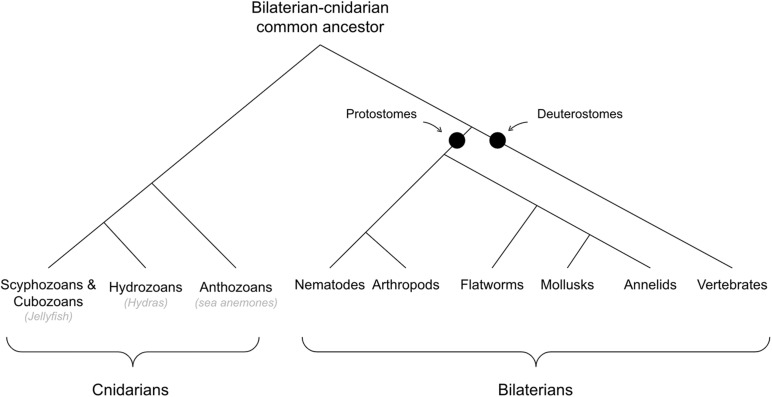
Cladogram of bilaterians and cnidarians.

See Figure 2 for cladogram of bilaterian-cnidarian divergence.

### Hypothesis #1: “Taxis Navigation” Emerged in Early Bilaterians

#### Taxis Navigation in Bilaterians (In-Group Condition)

The predominant navigational and hunting strategy across bilaterians is to navigate toward food and away from danger. Taxis navigation is the navigational strategy of simply turning *toward* or *away* from specific stimuli (e.g., chemotaxis, phototaxis, and thermotaxis). Even early diverging bilaterians, including those thought to be model organisms for urbilateria, such as *Caenorhabditis elegans* and flatworms, demonstrate taxis navigation. Both species climb sensory gradients to approach or avoid various stimuli (Pearl, 1903; Larsch et al., 2015).

There are two notable features of such taxis navigation in these model organisms for urbilateria: cross-modal integration and valence. Cross-modal integration describes the ability to integrate competing input across modalities to decide whether to approach or avoid a cue. For example, the decision by *C. elegans* to cross a copper barrier (which is aversive) to get to food on the other side is sensitive to the strength of the food smell relative to the concentration of copper (Hobert, 2002; Ishihara et al., 2002). Further, flatworms will navigate toward a food cue even if there is aversive light at the same source. But if made bright enough, flatworms will no longer travel all the way to the food source; and the brighter the light, the further from the food source they will end up. This type of integrated decision making was also shown across numerous modalities such as mechano-sensation and thermo-sensation (Inoue et al., 2015).

Valence refers to the ability to change decisions based on internal states, hence representing an ability to make decisions depending on what an organism needs. The concept of valence modulated by internal states is a foundational component of motivation and learning theories of behavior (Balleine, 2001, 2018; Berridge, 2019). For example, In *C. elegans*, food smells that trigger approach when hungry often have no effect when well fed (Davis et al., 2017). Further, some cues like carbon dioxide, which can signal both food as well as predators, shift from attractive when hungry to aversive when well fed (Rengarajan et al., 2019).

The neural substrates of taxis navigation, valence, and cross-model decision making are relatively well understood in *C. elegans*. The sensory neurons within *C. elegans* seem to encode valence directly: many of the sensory neurons in *C. elegans* are directly modulated by neuropeptides that signal internal states such as hunger or stress, and thereby provide a likely substrate by which behavior is sensitive to internal states (Chao et al., 2004; Chalasani et al., 2010; Sengupta, 2013; Guillermin et al., 2017; Rengarajan et al., 2019). In this sense, these sensory cells can be interpreted as primitive versions of valence neurons, with some responsive to positive valence stimuli (positive valence neurons), and others responsive negative valence stimuli (negative valence neurons). Cross-modal integration seems to occur through input to common neural circuits that control locomotion: there are command interneurons in the brain of *C. elegans*, which control turning by modulating locomotor central pattern generators (Garrity et al., 2010). One set of command interneurons biases movement toward *forward* locomotion whereas the other seems to bias movement toward *turning*. Different groups of sensory cells selectively target different sets of these command interneurons. The positive valence neurons in *C. elegans* that activate in response to the onset of food smells stimulate interneurons that trigger forward locomotion and suppress turning (Chalasani et al., 2007). On the other hand, negative valence neurons that activate in response to aversive stimuli such as copper, predator smells, or noxious heat, stimulate interneurons that trigger turning (Kimata et al., 2012). In general, these forward and turning neurons exhibit lateral inhibition of each other (Wakabayashi et al., 2004; Li et al., 2014).

#### Taxis Navigation in Non-bilaterian Metazoans (Out-Group Condition)

Sea anemones are the extant cnidarians most likely to resemble early cnidarians: they are believed to have diverged earliest in cnidarian evolution, resembling early cnidarians in the fossil record (Yuan et al., 2011), and they resemble the developmental polyp stage that all cnidarians go through (Harris, 1990; Hinde, 1998). The hunting strategy of sea anemones is primarily one of waiting for food to come to them, and catching prey with their tentacles (Ruppert et al., 2004).

Medusae cnidarians (such as jelly fish) likely evolved later than polyp cnidarians (such as sea anemones) and admittedly do spend most of their time moving. However, even medusae do not show active hunting toward food. Rather, they have various reflexive movement routines that drive locomotion in general. For example, medusae swimming seems to be simply driven by orienting their heads in the direction of current to ensure staying in the same general location (Fossette et al., 2015). While chemical cues such as those for food in medusae change the overall speed of locomotion (Matanoski et al., 2001), and the sensitivity of the feeding reflex (Pantin, 1935; Batham and Pantin, 1950a; Ewer and Fox, 2009), it doesn’t seem to broadly drive medusa navigation. Medusae do indeed swim up and down vertically in a Lévy walk-like manner, (Hays et al., 2011) but this functions to maximize the likelihood of encountering food, not to actively pursue it.

Most cnidarians do not even show immediate reflexive escape responses. Cnidarians often have a retraction reflex, but this doesn’t drive locomotion in any specific direction (Batham and Pantin, 1950a). Some jellyfish do exhibit escape swimming, but most do not, which implies such behavior evolved independently and is not a general feature of early Cnidaria (Mackie, 2004). Instead of rapid escape responses, repeated aversive stimuli seem to toggle cnidarians between different broad phases of reflexive locomotion. Repeated aversive stimuli applied to the sea anemone will increase their likelihood to begin crawling and change location (Batham and Pantin, 1950a). But this is often triggered some time later after the stimulus, sometimes even within the next night cycle many hours later (Batham and Pantin, 1950a). Further, it is also unclear whether movement is reliably in the opposite direction of the aversive stimulus (Batham and Pantin, 1950b). In fact, often the direction of relocation seems to be driven by factors irrelevant to the aversive stimulus, such as simply moving in the direction of increased elevation (Batham and Pantin, 1950b). Sea anemones have also been shown to change location in this undirected manner if temperatures rise too high (Sund, 1958).

Even sexual reproduction in cnidarians is, in general, not one that requires navigating toward mates or even interacting directly with them. Instead, pheromone and light cues trigger a coordinated “spawning” whereby gametes are released into the sea, and hence mating between nearby cnidarians is made possible (Hinde, 1998).

Taken together, it seems to be the case that although stimuli can impact overall movement arousal in cnidarians, they do not, in general, exhibit directed taxis navigation. That said, there are a few exceptions where cnidarians do show directed navigation toward or away from stimuli. For example, hydra specifically orient their bodies and navigate directly toward light sources (Wilson, 1891; Haug, 1933). And box jellyfish can use eye spots on their head to avoid obstacles when swimming through a tank (Garm et al., 2007). However, this obstacle avoidance seems to use lens-like eyes that are generally agreed to have evolved independently, and is unlikely to represent navigational strategies of the early cnidarian-bilaterian common ancestor (Nilsson, 2013; Bosch et al., 2017). Sea anemones have also been found to move specifically in the direction of light (Parker, 1916), but this has been shown to be independent of their own visual apparatus and driven by cues of nearby amoebae – sea anemones without these symbiotic amoebae fail to navigate toward or away from light (Pearse, 1974; Foo et al., 2019).

The neural mechanisms for navigation are also different between Cnidaria and Bilateria. Locomotion in Cnidaria seems to operate via a completely independent reflexive circuit. For example, if you remove the oral disk of a sea anemone, and leave only the bottom of the animal, they still navigate as regularly and normally (Batham and Pantin, 1950b).

Larvae of earlier diverging metazoans, such as sponges, show taxis navigation (Wapstra and van Soest, 1987; Woollacott, 1993; Leys and Degnan, 2001; Leys et al., 2002), even though their adult forms show no such behavior. However, this taxis navigation uses completely different mechanisms from that of bilaterians: ciliated cells along the back of the larvae become rigid in response to various cues as with single cellular taxis navigation. The mechanism for how this response is coordinated across cells is not yet clear but may simply leverage independent photoreceptors within each of these ciliated cells directly.

Adult forms of ctenophores (comb jellies) move through ciliated pumping as well and may represent an adult form metazoan with taxis navigation coordinated through neurons. However, how well extant ctenophores represent early metazoans is unclear, with meaningful evidence indicating that ctenophores independently evolved many features of nervous systems (Ryan, 2014; Moroz, 2015; Moroz and Kohn, 2016; Liebeskind et al., 2017). If true, their basis of taxis navigation would not be indicative of early metazoans before the cnidarian-bilaterian last common ancestor.

#### Possible Adaptive Function(s) of Taxis Navigation in Early Bilaterians (Stem-Group Condition)

Fossil records have found evidence of small worm-like bilaterians navigating the microbial mats of the sea floor in the Ediacaran period (Chen et al., 2013). Such taxis navigation would likely have enabled these early bilaterians to navigate chemical gradients more efficiently and effectively on the Ediacaran seafloor in search of carcasses or microbial patches.

Taxis navigation is present within single-celled organisms and is clearly a fundamental feature of cellular navigation. But evidence suggests that taxis-navigation in early bilaterians was the first time that taxis-navigation was implemented in the substrate of neurons and muscles, as opposed to cellular cilia. The cilia-based navigation of sponge or cnidarian larvae may have been limited in its ability to integrate competing inputs, limited in their ability to incorporate global need states, and may not have scaled well to larger organisms. In contrast, this neuron-based implementation of taxis-navigation in early bilaterians came with the crucial features of integration (balancing competing stimuli to make a single cross-modal decision) as well as valence (modulating those decisions by internal states), while potentially also enabling taxis navigation in larger morphologies.

#### Conclusion

Taken together, evidence across the in-group, out-group, and stem-group conditions is generally supportive of the hypothesis that taxis navigation (with neurons and muscles) emerged in early bilaterians, and was not present, at least with the same faculties, in the cnidaria-bilaterian last common ancestor. First, many, if not all, early diverging bilaterians show taxis navigation using neurons and muscles with the features of valence and cross-modal integration (in-group condition). Second, there is sparse evidence of taxis navigation in non-bilaterian eumetazoans, with many showing no such behavior (out-group condition). And third, the ecological strategy of early bilaterians was likely to actively pursue food, whereas for earlier diverging eumetazoans, possibly resembling sea anemones, it seems more likely to have been one of waiting for food to come to them – the latters strategy being one where taxis-navigation would have been much less adaptive (stem-group condition).

### Hypothesis #2: Associative Learning Emerged in Early Bilaterians

#### Associative Conditioning in Bilaterians (In-Group Condition)

Associative learning, both classical and instrumental, has been shown across Bilateria, even those that diverged very early such as mollusks (Hawkins et al., 1989), flatworms (Prados et al., 2012), and nematodes (Ardiel and Rankin, 2010).

Consistent with the idea that associative learning across Bilateria have common evolutionary roots, associative learning across protostomes and deuterostomes shares a broad set of common features - including latent inhibition, overshadowing, blocking, second order conditioning, and trace conditioning. Latent inhibition has been shown in honeybees (Abramson and Bitterman, 1986; Chandra et al., 2000, 2010; Sandoz et al., 2000; Ferguson et al., 2001; Fernández et al., 2009; Fernandez et al., 2012), mollusks (Loy et al., 2006), fish (Mitchell et al., 2011), goats (Lubow and Moore, 1959), and rats (Ackil et al., 1969; Boughner and Papini, 2006). Overshadowing and blocking have been observed in flatworms (Prados et al., 2012), honeybees (Couvillon and Bitterman, 1989; Smith and Cobey, 1994; Couvillon et al., 1997; Smith, 1997), mollusks (Sahley et al., 1981; Colwill et al., 1988; Loy et al., 2006; Acebes et al., 2009), rats (Kamin, 1968; Prados et al., 2013), humans (Arcediano et al., 1997; Prados et al., 2013), rabbits (Merchant and Moore, 1973), and monkeys (Cook and Mineka, 1987). Second order conditioning has been observed in mollusks (Hawkins et al., 1998; Loy et al., 2006), rats (Lay et al., 2018), and monkeys (Cook and Mineka, 1987). Further evidence for the idea that such learning features are foundational to old neural circuits, and not more advanced ones, these features of associative learning can be seen even in spinalized rats (rats that only contain a spinal cord) (Illich et al., 1994).

Further, the biological mechanisms for associative learning across bilaterians is incredibly similar. All bilaterians use very similar presynaptic and postsynaptic mechanisms for learning. For example, both invertebrates and vertebrates use cAMP as well as NMDA and AMPA receptors in learning processes (Kandel, 2001, 2006; Dubnau et al., 2002; Glanzman, 2010; Hawkins and Byrne, 2015). Neuromodulators such as dopamine and serotonin are involved in gating both presynaptic and postsynaptic learning processes across both vertebrates and invertebrates. The importance of such neuromodulators in learning has been shown in invertebrates such as crickets (Hammer and Menzel, 1998; Farooqui et al., 2003; Vergoz et al., 2007), fruit flies (Burke et al., 2012; Liu et al., 2012), honeybees (Hammer and Menzel, 1998; Farooqui et al., 2003; Vergoz et al., 2007), and *C. elegans* (Kusayama and Watanabe, 2000; Qin and Wheeler, 2007). Further, mechanisms for some forms of trace conditioning also seem to have common neurobiology whereby traces decay after synaptic discharge, and subsequent third factors such as dopamine signaling trigger synaptic weight changes (Cassenaer and Laurent, 2012; Dylla et al., 2013).

#### Associative Conditioning in Non-bilaterian Metazoans (Out-Group Condition)

Associative learning has for the most part not been observed in non-bilaterian metazoans. Attempts to show associative learning in cnidarians have shown negative results (Rushforth, 1973; Torley, 2009). I am aware of only one report of associative learning in Cnidaria, where a sea anemone was reported to learn to contract tentacles in response to a light that predicted shock (Haralson, 1975). Subsequent results have not replicated this, and others who have reviewed the available literature have similarly concluded that Cnidaria do not exhibit associative learning (Ginsburg and Jablonka, 2019).

#### Possible Adaptive Function(s) of Associative Conditioning in Early Bilaterians (Stem-Group Condition)

The adaptive benefits of associative conditioning are straightforward to imagine in the ecological niche of an early bilaterian animal of the Ediacaran. The ability to learn to associate certain cues, such as light, with food or predators, would have been useful in biasing taxis navigation toward safer and more food-rich areas.

#### Conclusion

Taken together, evidence across the in-group, out-group, and stem-group conditions is supportive of the hypothesis that associative learning emerged in early bilaterians. First, associative learning is observed even in very early diverging bilaterians (in-group condition). Second, there are numerous negative results of associative learning in non-bilaterians eumetazoans (out-group condition). And third, if the ecological niche of early bilaterians was to actively pursue food, while for earlier eumetazoans it was to wait for food to come to them, then associative learning would have been uniquely adaptive for early bilaterians, who were regularly making important navigational decisions (stem-group condition). The hypothesis that associative learning emerged in early bilatearans has also been proposed by others (Ginsburg and Jablonka, 2021).

## Behavioral Abilities That Emerged in Early Vertebrates

**FIGURE 3:**
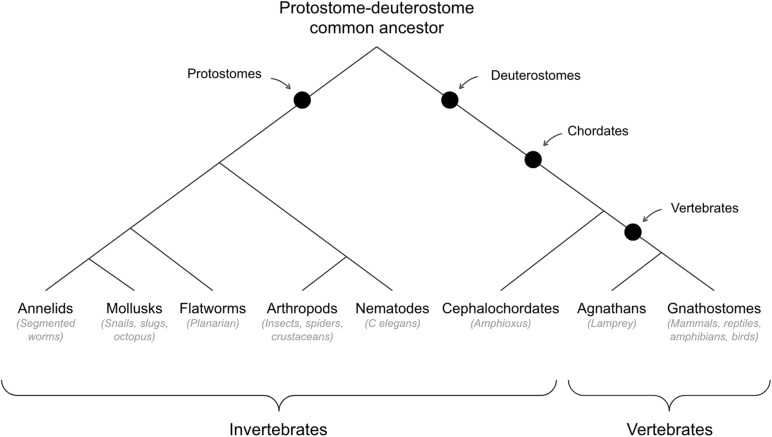
Cladogram of vertebrate-invertebrate divergence.

See Figure 3 for cladogram of vertebrate-invertebrate divergence.

### Hypothesis #3: Map-Based Navigation Emerged in Early Vertebrates

#### Map-Based Navigation in Vertebrates (In-Group Condition)

Many diverse vertebrates, including those that diverged early such as fish (Burt de Perera et al., 2016), reptiles (Wilkinson and Huber, 2012; Broglio et al., 2015), turtles (López et al., 2001), amphibians (Phillips et al., 1995), and tortoises (Wilkinson et al., 2007) show incredibly sophisticated mapping abilities (Rodríguez et al., 2002a) – capable of learning un-cued locations and capable of flexibly generating new navigation routes. For example, fish can remember specific locations in 3-dimensional space (Karnik and Gerlai, 2012; Lucon-Xiccato and Bisazza, 2017; Wallach et al., 2018) and can generate a correct novel path to specific goal locations from many different starting places (Brown, 2015). Fish can learn a place preference for locations that avoid pain, which persists even when visual cues are switched and changed (as long as background place cues are kept constant), demonstrating a stable representation of a location (Valente et al., 2012). Fish can also use spatial maps for navigation even when they are at odds with taxon-based cues (Katie, 2015). Fish can latently learn a map of a maze where no locations have been rewarded, and one-shot generate the correct path to an observed goal location (Gómez-Laplaza and Gerlai, 2010). Reptiles can generalize learning about navigating around detours quite well too (Wilkinson and Huber, 2012).

As further evidence that spatial maps emerged in early vertebrates, spatial memory across vertebrates use similar circuitry, and that circuitry is unique to vertebrates. It is known that the hippocampus is the locus for spatial maps in mammals (O’Keefe and Dostrovsky, 1971; Ranck, 1973). Hippocampal lesions lead to failures in many tests of map-based navigation, while leaving cue learning intact (Broadbent et al., 2004; Clark et al., 2005). Further, recording studies have shown grid cells, head direction cells, and place cells within various regions of the hippocampal complex, demonstrating a very sophisticated model of allocentric space within the hippocampus (O’Keefe, 1976; Fyhn et al., 2004; Hafting et al., 2005; Sargolini et al., 2006).

Evidence suggests that the pallium of non-mammalian vertebrates contains a homologous region to the hippocampus, sharing similar circuitry, embryonic origins, and genetic markers (Rodríguez et al., 2002a, b; Gupta S. et al., 2012; Abellan et al., 2014; Tosches et al., 2018). In reptiles the hippocampal homolog is the medial pallium, while in teleost fish it is the dorsolateral telencephalon (Rodríguez et al., 2002a, b). Lesioning these areas in goldfish or turtles similarly prevents allocentric spatial learning while leaving cue learning intact (López et al., 2003; Durán et al., 2010; Broglio et al., 2015). Recording studies of these hippocampal homologs in non-mammalian vertebrates such as fish similarly show such head direction cells, edge detection cells, velocity cells, and place cells (Vinepinsky et al., 2018, 2020). And lastly, the circuitry of these hippocampal homologs in non-mammal vertebrates is similar to the circuitry of the hippocampus in mammals (Giassi et al., 2012; Fotowat et al., 2019).

Some evidence suggests that the basal ganglia, a structure that emerged in the first vertebrates (Grillner and Robertson, 2016), is also necessary for map-based navigation (Goodroe et al., 2018). Interactions between the hippocampus and basal ganglia seem to be necessary for the development of conditioning place preference or conditioned place aversion (Ito et al., 2008). Some have suggested that there may have precursors of the basal ganglia in the bilaterian common ancestor, given possible homology to arthropod central complex (Strausfeld and Hirth, 2013), but this is currently unclear.

#### Map-Based Navigation in Invertebrates (Out-Group Condition)

Note that I will use the label invertebrate as a shorthand for non-vertebrate bilaterians.

Taxon navigation refers to learning to take specific actions at specific cues. Map-based navigation, on the other hand, is when an animal learns a spatial map of an environment enabling them to calculate new routes that have never been tried before to get to a familiar location, and to learn about locations, even if individual cues are no longer present or have moved. Many reviews of navigation amongst even sophisticated invertebrates have concluded that their navigational strategy is exclusively one of taxon navigation, not of map-based navigation (Wehner et al., 1996; Walker, 1997; Benhamou et al., 1990).

Invertebrates fail at tests designed to evaluate their ability to generate maps. For example, if ants are put in an experimental condition where they must have an outbound route away from their nest to get to food, and a different inbound route back to the nest, they will readily learn how to follow this exact route. The question is – are they learning simply to turn at specific cues, or do they understand the map of their environment? A simple test is to place an ant trying to get back to its nest on the outbound path; if the ant understood a map of the environment, it would simply turn around and go right back (this shows flexible calculation of new routes to a target location) - but the ant doesn’t do this, it becomes lost (Wehner et al., 2006). Further, if you change a single cue in an environment as an ant navigates (but leave other cues indicative of the map of the environment intact), it can completely disrupt their navigation (Cheng, 2012).

In another experiment, it was shown that a bee can learn to push a cap in the middle of a plate to find food underneath. If you then put a different shaped cap in the center with food underneath, and put the old cap on the far end of the plate, the bee will simply go to the original cap. It must completely re-learn to push the new cap: the bee never learned to associate a location with the reward (Abramson et al., 2016). In contrast, honeybees can learn to navigate through a very complex mazes using taxon navigation, such as by learning to turn right or left at specific-colored cues (Zhang et al., 2000). Early diverging bilaterians such as *C. elegans* and planarians also do not navigate using spatial maps and instead use basic taxis mechanisms (Pearl, 1903; Luersen et al., 2014; Larsch et al., 2015).

There have been reports and suggests that a few select invertebrates have been shown to build map-like memories (Boles and Lohmann, 2003; Menzel et al., 2005, 2011). But this is still with considerable controversy, with others claiming that the ability to navigate from novel locations in these invertebrates is in fact just a sophisticated path integration mechanism and includes no map-like representations (Wehner et al., 1996; Lehrer, 1997a, b). This is further complicated by the fact that many impressive abilities of insects seem to emerge from mushroom bodies (Perry et al., 2013; Cope et al., 2018), a cortex-like structure which is believed to have evolved independently (Farris, 2008). As such, any behavioral abilities emerging from the mushroom bodies are more likely a case of independent evolution and not representative of the protostome-deuterostome last common ancestor.

#### Possible Adaptive Function(s) of Map-Based Navigation in Early Vertebrates (Stem-Group Condition)

Fossils of *Haikouichthys* spp., believed to be some of the earliest vertebrate species (Shu, 2003; Shu et al., 2003, 2009), have been dated to the early Cambrian (Zhang et al., 2001; Shu et al., 2003). The ecological niche of these early vertebrates is unknown, but relative to the huge fossils of presumably predatory arthropods from the Cambrian, we can speculate that there was strong pressure to avoid predation. Map-based navigation may have been a technique that these early vertebrates used to quickly swim to safety in response to predators as well as how to avoid dangerous locations, both useful adaptations to avoid predation. Some evidence suggests that early vertebrates may have evolved in shallow waters (Griffith, 1994; Sallan et al., 2018), which would have contained many more landmarks than in the open sea, perhaps making map-based navigation uniquely useful.

#### Conclusion

Taken together, evidence across the in-group, out-group, and stem-group conditions is generally supportive of the hypothesis that map-based navigation emerged in early vertebrates. First, map-based navigation is observed even in early diverging vertebrates and the neural substrates of map-based navigation in vertebrates seem to be structures that emerged only in early vertebrates (in-group condition). Second, there are numerous negative results of map-based navigation in invertebrates, especially those thought to be model organisms for the protostome-deuterostome last common ancestor such as *C. elegans* or planarians; and those invertebrates that do show some potential evidence of map-based navigation, such as arthropods, seem to do so in independently evolved substrates, such as the mushroom body (out-group condition). And third, map-based navigation would have been adaptive in the predatory shallow water environment of early vertebrates.

### Hypothesis #4: “Interval Timing” Emerged in Early Vertebrates

#### Interval Timing in Vertebrates (In-Group Condition)

Even vertebrates that diverged very early, such as fish, show a remarkable ability to learn the timing of events. For example, goldfish and zebrafish can remember the exact time interval between a cue and a shock occurring and will selectively speed up to escape right before the shock occurs (Drew et al., 2005; Lee et al., 2010; Meck et al., 2012). Such interval timing is shown across vertebrate phyla, including in fish (Sumbre et al., 2008), birds (Bateson and Kacelnik, 1997; Ohyama et al., 1999; Buhusi et al., 2002), non-human primates (Gribova et al., 2002), and mice (Roberts and Church, 1978; Gallistel et al., 2004; Buhusi et al., 2005). Although some vertebrates do struggle with such tasks (Grossmann, 1973; Kleniginna and Currie, 1979; Laurent and Lejeune, 1985; Lejeune and Wearden, 1991).

Interval timing also seems to be implemented by uniquely vertebrate brain regions, namely, the striatum, hippocampus, and cerebellum. Many models of how interval timing is implemented in the brain place the striatum as the locus of interval timing (Matell and Meck, 2000, 2004; Meck and Benson, 2002). Additional models also incorporate the hippocampus into these timing mechanisms (MacDonald et al., 2011; Oprisan and Buhusi, 2013; Oprisan et al., 2018; Rolls and Mills, 2019; Shimbo et al., 2021). Lesions of the hippocampus in humans and rats disrupt learning on interval timing tasks (Meck et al., 1984, 1987; Melgire et al., 2005; Balci et al., 2009; Yin and Meck, 2014). Damage to the striatum in the basal ganglia leads to even more severe interval timing performance (Malapani et al., 1998, 2002). The results on lesions in both structures are specific to the timescale of hundreds of milliseconds to seconds. When it comes to millisecond-based timing, and perhaps also absolute timing in general, the cerebellum, seems to be essential (Ivry and Spencer, 2004).

#### Interval Timing in Invertebrates (Out-Group Condition)

As noted in other reviews of invertebrate learning and behavior, invertebrates consistently show an inferior perception of time to vertebrates, if any perception of time at all (reviewed in Abramson and Wells, 2018). Invertebrates struggle to learn and predict the specific times at which events will occur and differentiate between different timings (Abramson and Feinman, 1990; Balci, 2015). Honeybees show an inability to tell the difference between stimuli separated by 15, 30, 60, or 120 s (Craig et al., 2014). They also do not increase responses as the time of stimulus presentation approaches, suggesting they do not anticipate an event based on its timing (Abramson and Boyd, 2001; Craig et al., 2014). Similar results have been shown for crabs (Abramson et al., 1988; Abramson and Feinman, 1990; Balci, 2015). There are some studies that have shown evidence of interval timing in bees (Boisvert and Sherry, 2006), but the methodology of analysis has been questioned because it only demonstrated group-average differences and did not report on individual performance (Abramson and Wells, 2018).

#### Possible Adaptive Function(s) of Interval Timing in Early Vertebrates (Stem-Group Condition)

If early vertebrates experienced strong predation pressures from Cambrian arthropods and lived near shores with many landmarks of underwater rocks, plants, and even corals, then interval timing may have yielded certain adaptive benefits. For example, it would have been adaptive to learn the timing between faraway predator cues and their actual arrival. Further, as seen in the next section, it is possible that interval timing was a prerequisite for omission learning, which has many benefits for flexible and robust predictions, which would have been useful in avoiding predation.

### Conclusion

Taken together, evidence across the in-group, out-group, and stem-group conditions is supportive of the hypothesis that the ability to learn the specific timing between events emerged with early vertebrates. First, interval timing has been observed in early diverging vertebrates and the neural substrates of interval timing seem to be structures that emerged in early vertebrates (in-group condition). Second, there are numerous negative results of interval timing in invertebrates (out-group condition). And third, it is straightforward to imagine the adaptive benefits of interval timing in the predatory environment in which early vertebrates evolved.

### Hypothesis #5: Omission Learning Emerged in Early Vertebrates

#### Omission Learning in Vertebrates (In-Group Condition)

Another difference in the observed behavioral abilities between extant vertebrates and invertebrates is omission learning. The standard paradigm wherein omission learning is evaluated is as follows. First, a cue (e.g., a light) is paired with a shock, via classical conditioning. Second, trained animals are given the option to prevent the shock after the cue is presented, such as by moving to one side of the cage or by pushing a lever. These actions either terminate the cue and prevent the shock or prevent the shock without terminating the cue. Three levels of understanding can be tested in this paradigm. Level one is Pavlovian learning: animals can learn the predictive relationship between the cue and the shock. Level two is offset learning: animals can learn to repeat behaviors that terminate the cue that has been paired with pain. Level three is omission learning: in response to a cue that reliably predicts pain, animals can learn to repeat behaviors that lead to the omission of predicted pain, even if the cue is *not* terminated.

Many vertebrates, including dogs (Cole and Wahlsten, 1968), mice (Kamin, 1957; Avcu et al., 2014), and fish (Portavella, 2004; Vindas et al., 2012, 2014) demonstrate the ability to learn from omission. A key neural substrate of omission learning in vertebrates is dopamine reward prediction errors: tonic dopamine can pause during negative valence and burst during positive valence. A leading model of how this works is that striosomes in the vertebrate striatum learn to predict and time activations of dopamine and directly inhibit dopamine neurons (Brown et al., 1999). In other words, striosomes filter out predicted dopamine activations, and hence trigger opposing dopamine activations when an unconditioned stimulus is omitted (e.g., positive valence when pain is omitted, or negative valence when food is omitted). Evidence suggests that this omission learning circuitry is shared by all vertebrates, including fish. In zebrafish for example, there are reward prediction errors during omission throughout its brain (Li, 2012; Cheng et al., 2014). And further, if you inactivate the habenula in zebrafish during avoidance learning, it strongly biases them from avoiding, and they become stuck only freezing, as you would expect if avoidance learning were driven by the rewarding aspects of omitted pain (Agetsuma et al., 2010).

#### Omission Learning in Invertebrates (Out-Group Condition)

Invertebrates seem, for the most part, to only learn from conditioned cue offsets, and seem incapable of learning from solely the omission of an unconditioned stimulus (reviewed in Abramson and Wells, 2018). In other words, invertebrates seem to only operate at “level two” in the above description. The key test to tell the difference between offset and omission learning is whether an animal’s behavior is reinforced if it results in the omission of an aversive unconditioned stimulus, even when the conditioned cue (which normally predicts the unconditioned stimulus) is not terminated by said behavior. With omission learning this omission without termination will still be reinforcing, with only offset learning it will not. This general type of avoidance test has been performed across many invertebrates, including crabs, ants, and honeybees (Abramson et al., 1988; Abramson and Wells, 2018), all consistently demonstrating the inability to learn from omission. Consistent with this, flies have been shown to struggle to respond appropriately when expected cues are omitted (Wenner and Wells, 1990; Sanderson et al., 2013).

The observation that invertebrates fail at omission learning is somewhat perplexing because many observed features of learning in invertebrates, such as blocking, are best explained by the Rescorla–Wagner (RW) model, whereby learning is gated by surprise (Rescorla and Wagner, 1972). If invertebrates gate learning by surprise, then one would expect an explicit prediction error signal, which should enable invertebrates to learn from omission. However, a more detailed examination of the neural substrates of associative learning in invertebrates and vertebrates helps illuminate why this difference exists, and why although both vertebrates and invertebrates learn using an Rescorla–Wagner learning rule, invertebrates still can’t learn from omissions.

**FIGURE 4:**
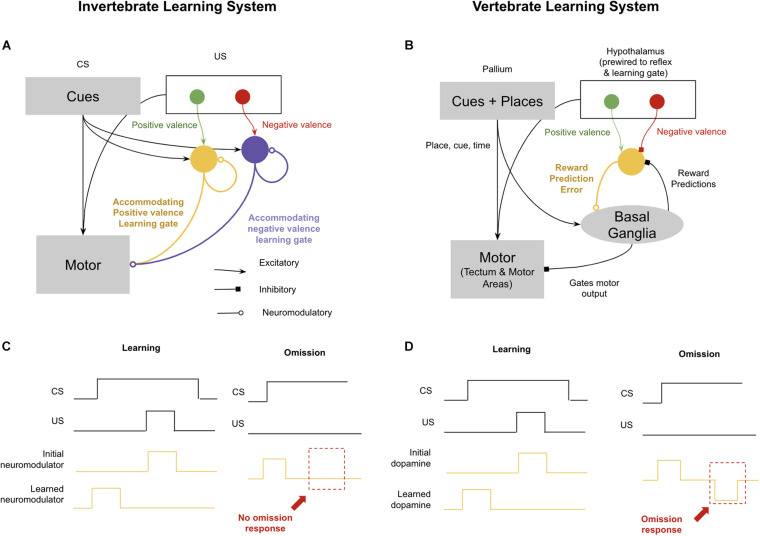
Difference in reinforcement learning between (most) invertebrates and vertebrates. Invertebrates implement reinforcement learning through accommodating neuromodulators, each of which modulates different valences and reflexes. This enables complex learning, but not omission learning. In contrast, vertebrates use dopamine to encode both positive and negative reward prediction errors, which enables learning through omission. See text for details. **(A)** Schematic of invertebrate reinforcement learning system. **(B)** Schematic of vertebrate reinforcement learning system. **(C)** Example of associative learning in accommodating neuromodulator response to conditioned stimulus (CS) predictive of unconditioned stimulus (US). Omission example (right) demonstrates lack of inverse response to omitted US. **(D)** Example of associative learning in dopamine reward prediction error. Omission example (right) demonstrates presence of omission response when US is omitted (after learning).

The standard invertebrate circuits for conditioning are those described elegantly in *Aplysia* spp. by Kandel (1979, shown in Figure 4). During the pairing of a conditioned stimulus and an unconditioned stimulus, sensory neurons for the conditioned stimulus potentiate their synapses with neuromodulatory neurons. Next time the conditioned stimulus occurs, they can activate neuromodulatory neurons directly. It is then believed that these neuromodulatory neurons are accommodating, meaning that if they first burst sufficiently in response to the conditioned stimulus, they will be unresponsive to an unconditioned stimulus occurring shortly thereafter. This means that during an animal’s experience with a conditioned stimulus and then an unconditioned stimulus, the neuromodulatory burst will shift forward to the conditioned stimulus and away from the unconditioned stimulus. The consequence of this is a simple version of the Rescorla–Wagner rule that will generate the effects of blocking, second-order conditioning, and overshadowing (Hawkins and Kandel, 1984; Hawkins et al., 1998). This circuit can even enable offset learning, if you assume that negative valence conditioned stimulus’ inhibit positive valence neuromodulators, and their offset drives rebound excitation. But what this primitive circuit will fail to learn is when something is predicted but never occurs. There is no notion of timing in this circuit, and hence if a conditioned stimulus is active, predicting a shock 5 s later, and the conditioned stimulus remains but the shock never occurs, nothing in this circuit will encode a prediction error in that omission. The relationship between omission learning and the emergence of time perception is likely not a coincidence. Without the ability to accurately encode interval timing, learning from omission would be very difficult: to assess when something has been omitted, an animal must not only predict *what* will happen, but also *when* it will happen.

#### Possible Adaptive Function(s) of Omission Learning in Early Vertebrates (Stem-Group Condition)

As shown in temporal difference learning, the ability to learn from predicted events that do not occur leads to much more robust and flexible learning (Sutton, 1988). Hence, it is reasonable to speculate that omission learning enabled early vertebrates to learn more effectively to predict food locations and predator behaviors.

#### Conclusion

Taken together, evidence across the in-group, out-group, and stem-group conditions is supportive of the hypothesis that the ability to learn from omissions emerged in early vertebrates and did not exist in brains beforehand. First, omission learning has been demonstrated across vertebrates and seems to be implemented in neural structures that are emerged in early vertebrates (in-group condition). Second, multiple negative results have been demonstrated in invertebrates (out-group condition). And third, omission learning would have offered many adaptive benefits to early vertebrates, as this ability is a key feature of temporal difference reinforcement learning.

## Behavioral Abilities That Emerged in Early Mammals

**FIGURE 5:**
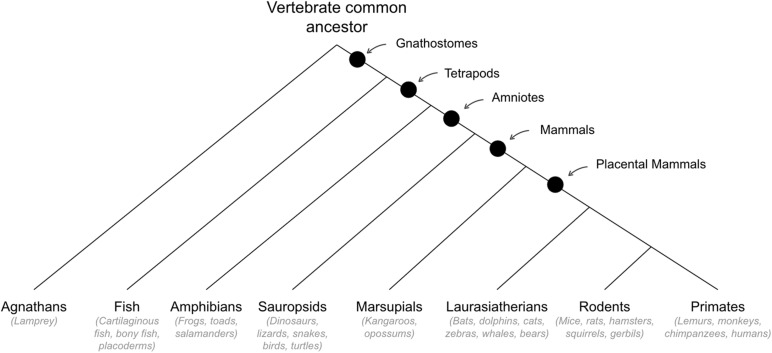
Cladogram of mammal divergence.

See Figure 5 for cladogram of mammal divergence.

### Hypothesis #6: Vicarious Trial and Error Emerged in Early Mammals

#### Vicarious Trial and Error in Mammals (In-Group Condition)

It has long been observed that mice navigating a maze will occasionally pause at choice points and toggle their head back and forth – this behavior has been called vicarious trial and error (VTE) and it has been interpreted as the mice considering their options (Muenzinger and Gentry, 1931; Tolman, 1939, 1948; Tolman and Minium, 1942). VTE shows several interesting features. First, animals perform VTE selectively when decisions are hard, such as when in the early stages of learning about a maze (Tolman and Minium, 1942), when the difference between the outcomes is small (Tolman, 1939; van der Meer et al., 2010), when starting in different places (Gardner et al., 2013; Schmidt et al., 2013), when reward contingencies change (Johnson and Redish, 2007; Steiner and Redish, 2012; Regier et al., 2015), or when there is conflict between evidence (Schmidt et al., 2013). When decisions are not difficult, such as when an animal can learn simple procedural strategies, such as always turn left at this cue, then VTE goes away (Gardner et al., 2013). This VTE behavior has also been shown across the mammalian taxa, including in non-human primates and humans (reviewed in Redish, 2016). Such head turning behavior in humans has been shown to be predictive of superior performance (van der Meer et al., 2010).

Three structures are highly implicated in VTE behavior: the neocortex, the hippocampus, and the ventral striatum (VS). When rats perform VTE, the hippocampus replays the same path sequences of place cells that represent the routes to each goal, demonstrating that the animal is first simulating the path toward one goal, and then simulating the path toward the other, without actually moving (Johnson and Redish, 2007; Gupta A. S. et al., 2012; Wikenheiser and Redish, 2015). The preplay events are identified as sharp wave ripples in recording studies. These sharp wave ripples are not found in non-mammal vertebrates (Van Twyver and Allison, 1972; Hough and Bingman, 2004; Kahn et al., 2008; Rattenborg et al., 2011; Ben-Yishay et al., 2020; Vinepinsky et al., 2020) with the exception of birds, where they have been found in the avian hippocampus (a functional homolog of mammalian hippocampus) during sleep (Payne et al., 2020).

It has been hypothesized that this hippocampal preplay is initiated by the prefrontal cortex (Redish, 2016). Evidence for this can be seen in the fact that hippocampal disruption increases VTE behavior (Robbe et al., 2006; Bett et al., 2015), as if the animal is trying to simulate actions but struggling to successfully do so without an intact hippocampus. Consistent with this, hippocampal damage makes rats more impulsive in delayed gratification tasks (Cheung and Cardinal, 2005), as if unable to simulate the benefit of waiting. The prefrontal cortex is known to highly influence such goal-based decisions (Killcross and Coutureau, 2003; Sharpe and Killcross, 2015) and be required for goal related activity in the hippocampus (Ito et al., 2015; Spellman et al., 2015). Prefrontal cortex disruption impairs an animal’s ability to make hard decisions at choice points, implying disruption to VTE (Ragozzino et al., 1999). Specifically at choice points in mazes, rats exhibit entrainment between oscillatory activity in the hippocampus and prefrontal cortex (Benchenane et al., 2010; O’Neill et al., 2013; Spellman et al., 2015). Also supportive of the idea of prefrontal cortex involvement in VTE, mice with PFC lesions are still able to solve the Morris water maze task and navigate spatial maps normally (Poucet, 1989, 1990; Granon and Poucet, 1995), but show impaired behavior when the task is made exceptionally hard by making rats start from completely novel locations (Granon and Poucet, 1995). Lastly, prefrontal cortex is known to be engaged during planning in general (Redish, 2016).

Evidence suggests that the ventral striatum (VS) is the neural substrate whereby the outcome of the simulated options are evaluated. During VTE behavior, cells in the VS encode reward values of the goal outcome of each option (van der Meer and Redish, 2009; Steiner and Redish, 2014; Stott and Redish, 2014). Importantly, these are the same cells that become active during the receipt of actual rewards (Tremblay and Schultz, 1999; Nicola et al., 2004; Roitman et al., 2005; Padoa-Schioppa and Assad, 2006; Stott and Redish, 2014).

#### Vicarious Trial and Error in Non-mammal Vertebrates (Out-Group Condition)

To my knowledge, there have been no published evaluations of specifically VTE outside of mammals. Some suggestive evidence has been seen in birds, who have demonstrated the ability to plan their foraging paths ahead of time (Sulikowski and Burke, 2015), which is suggestive that birds can also engage in a form of VTE. However, for reasons that will be discussed more thoroughly in hypothesis #8, birds are poor model organisms for the brains of the amniote common ancestor; bird brains independently underwent substantial modification since the amniote common ancestor and as such are unlikely to be representative of the brains of early amniotes.

#### Possible Adaptive Function(s) of Vicarious Trial and Error in Early Mammals (Stem-Group Condition)

The early mammals of the Mesozoic period are believed to have been small nocturnal (Polyak, 1957; Jerison, 1973; Gerkema et al., 2013; Wu et al., 2017), arboreal (Fröbisch and Reisz, 2009; Luo et al., 2015; Meng et al., 2015) insectivores (Kielan-Jaworowska et al., 1979; Lofgren et al., 2004). They would have likely been under extreme predation pressure from the massive archosaurs of the Mesozoic. Their ecological niche was likely hiding in trees and burrows, only to emerge for the purpose of quickly hunting food. VTE may have been useful in deciding which path to take across branches to either get to nearby insects or to avoid nearby predators. Navigating tree branches with far eyesight presents unique challenges and evolutionary pressures that may not have been previously experienced to the same degree: namely, irreversible choices. As a small animal living in trees, you must plan your route well in advance. And it is likely you will very regularly experience novel branches. Additionally, computational models have found that the usefulness of planning is directly tied to visual range. Visual range in water is so poor that computational models suggest planning in water is barely useful at all (Mugan and MacIver, 2020), whereas on land, such planning is highly adaptive.

#### Conclusion

Taken together, evidence across the in-group, out-group, and stem-group conditions is generally supportive of the hypothesis that VTE emerged in early mammals. First, VTE has been well observed even in mammals that diverged well before the first primates and some of the key neural substrates of VTE emerged in early mammals (“in-group condition”). Second, VTE behavior has not been reported in non-mammal vertebrates, outside of possible evidence in birds (“out-group condition”). And third, VTE has been shown to improve decision making, which would likely have been adaptive to the small arboreal and nocturnal mammals in the Mesozoic. However, the lack of published negative results in non-mammal vertebrates renders this hypothesis tentative. More studies will have to be done to further support or refute this proposal.

### Hypothesis #7: Counterfactual Learning Emerged in Early Mammals

#### Counterfactual Learning in Mammals (In-Group Condition)

A hallmark of human intelligence is the ability to consider things that might have happened had we made a different past decision. Such counterfactual learning has been observed in rats, monkeys, and humans (Zhang et al., 2015). A test of counterfactual learning has called the restaurant row test. In such a test, rats can wait for a higher quality meal or take a smaller, lesser meal more quickly. Once a choice was made, they cannot reverse it. Two key results were found. First, rats that choose the worse deal tend to look back and glance at the room they skipped. Second, after experiencing a regret-inducing situation (where they took the smaller reward, but then saw had they waited, they would have gotten the better one), rats tend to wait longer at the high-cost option during the next trial (Lewis, 2014; Steiner and Redish, 2014). In a similar study it was shown that rats actively choose behavior to avoid these experiences of regret (Sweis et al., 2018). Counterfactual learning has also been shown in non-human primates, where if they are made to play rock paper scissors, selectively after losing, they show a strong bias toward playing the move that would have won the last round (Abe and Lee, 2011).

In the rat studies above, it was shown that neurons within the orbitofrontal cortex and VS represented the counterfactual action when rats looked back at their rejected option (Gilovich and Medvec, 1995). They further showed that the greater this representation of the counterfactual action in the orbitofrontal cortex and VS, the higher the likelihood rats would stay and wait for the counterfactual choice next time they were presented with the task. Note that, consistent with the idea that imagining an event or stimulus reactivates the same circuitry as experiencing the event or stimulus firsthand, across mammals it has been shown that neurons in the orbitofrontal cortex also encode the reward values (Padoa-Schioppa and Assad, 2006; Sul et al., 2010; Abe and Lee, 2011). This implies that counterfactual learning includes re-simulating the reward of the alternative option, much the same way VTE simulates different paths. Humans also show orbitofrontal activity during regret (Coricelli et al., 2005, 2007). In fact, humans with damage to the orbitofrontal cortex seem to be unable to experience regret (Camille et al., 2004).

#### Counterfactual Learning in Non-mammal Vertebrates (Out-Group Condition)

To my knowledge, there have been no published evaluations of counterfactual learning in non-mammals.

#### Possible Adaptive Function(s) of Counterfactual Learning in Early Mammals (Stem-Group Condition)

Counterfactual learning has numerous benefits as demonstrated in various machine learning models that leverage hindsight experience replay (Andrychowicz et al., 2017). In a situation with three or more possible actions, when a mistake is made and the correct action is obvious after the mistake, an animal without counterfactual learning will only learn to inhibit the chosen action, but not to reinforce the observed correct one.

#### Conclusion

Taken together, evidence across the in-group, out-group, and stem-group conditions is generally supportive of the hypothesis that counterfactual learning emerged in early mammals. First, counterfactual learning has been observed in relatively early diverging mammals, key neural substrates of counterfactual learning are structures that emerged in early mammals (in-group condition). Second, counterfactual learning has not been reported in non-mammal vertebrates (out-group condition). And third, counterfactual learning offers many learning benefits, which would have been adaptive to early mammals (stem-group condition). However, the lack of published negative results in non-mammal vertebrates makes this hypothesis tentative.

### Hypothesis #8: Episodic Memory Emerged in Early Mammals

#### Episodic Memory in Mammals (In-Group Condition)

A key test of episodic memory is whether an animal can answer an unexpected question about their own experience. The question must be unexpected because it requires the animal to inquire their own mind for the answer – if it is expected the behavior can simply be instrumentally associated with the past action via trace conditioning.

The ability to answer unexpected questions has been shown in mammals such as dogs (Fugazza et al., 2020), rats (Crystal, 2013), and non-human primates (Menzel, 1999). As an example, rats trained to get a reward differently depending on whether they recently experienced food or not, can be randomly asked this question throughout normal foraging and exploration. Rats can successfully report on whether they had just recently experienced food whenever they are unexpectedly asked this question (Crystal, 2013).

The neural mechanisms of episodic memory seem to be homologous across mammals. For example, in mammals, episodic memory uses the same neural circuitry as simulating the future (Suddendorf and Corballis, 1997; Schacter et al., 2007; Martin et al., 2011; Allen and Fortin, 2013). The general view is that the frontal cortex asks a question to the hippocampus, inquiring about some past event and the hippocampus contains a pointer to the contents of that event, which then reactivates the whole episodic memory within the neocortex (just like with VTE) (McClelland and Goddard, 1996; Eichenbaum et al., 2007). The neocortical representation of the retrieved memory is then transmitted to the frontal cortex and striatum for evaluation and action selection (Goldman-Rakic, 1996; Fuster, 2001; Ninokura et al., 2003; Eichenbaum and Fortin, 2009).

Various studies are consistent with this view of episodic memory. For example, in the one study whereby rats answered unexpected questions about their past experiences, the experimenters temporarily inhibited the hippocampus specifically during the moment rats were asked questions. What they found was that when rats answered *expected* questions (hence not requiring internal inquiry), hippocampal inactivation had no effect on performance. However, when the questions were unexpected, rats completely lost the ability to successfully inquire on their own episodic memory. This is suggestive of the idea that the hippocampus is specifically crucial for the reactivation of episodic memories. The hippocampus has been proposed to play the same role in human episodic memory (Wixted et al., 2018). Further consistent with this model of episodic memory, studies have shown that remembering past events reactivates the exact same cortical representations of the experience of it itself (O’Craven and Kanwisher, 2000; Pearson et al., 2015).

#### Episodic Memory in Non-mammal Vertebrates (Out-Group Condition)

Episodic memory of this form, whereby animals answer unexpected questions about their past, has also been shown in pigeons (Zentall et al., 2001, 2008; Singer and Zentall, 2007) and cephalopods (Billard, 2020). However, the neural mechanisms underlying episodic memory in these species seem to be non-homologous with the neural mechanisms in mammals. Episodic memory in mammals is highly dependent on the neocortex, a structure with which at least cephalopods have no homologous region.

Admittedly, some evidence suggests that the dorsal ventricular ridge of birds (which contain the nidopallium and mesopallium) and the neocortex of mammals both derive from the pallium of their shared amniote ancestor (Karten, 1969, 1997; Reiner et al., 2004; Dugas-Ford et al., 2012). These studies demonstrate that the dorsal ventricular ridge and neocortex share many features, including the subcortical structures they interact with, and the molecular properties of their neurons. Hence if episodic memories in mammals is dependent on the neocortex, perhaps this function was derived from the pallium of the amniote or even vertebrate last common ancestor and is not dependent on the unique features of neocortex. However, this interpretation is unconvincing for two reasons.

First, birds are a poor model organism for the brain of the amniote last common ancestor. Some have suggested that the dorsal ventricular ridge is not homologous with the neocortex and instead shares homology with the mammalian amygdaloid complex (Jarvis et al., 2005; Striedter, 2005). And even if the dorsal ventricular ridge does share homology with the neocortex, the dorsal ventricular ridge has completely distinct microcircuitry from the neocortex. The neocortex is organized into six layers, while the dorsal ventricular ridge is organized into clustered nuclei (Ulinski, 1983). The ontogeny of the dorsal ventricular ridge and the neocortex in mammals is also different (Jones and Levi-Montalcini, 1958; Striedter and Keefer, 2000; Dugas-Ford et al., 2012). Further, the pallial homolog of other extant amniotes such as non-bird reptiles, also have completely unique ontogeny and microcircuitry (Goffinet et al., 1986; Cheung et al., 2007). For example, turtles have a three layered cortex, instead of the clustered nuclei of the dorsal ventricular ridge, or the six layered neocortex. The turtle cortex is more like the three layered pallium of other non-amniote vertebrates, such as fish, than it is to the dorsal ventricular ridge of birds or the neocortex of mammals. This is thereby suggestive that many of the pallial homologs in birds and mammals have undergone substantial independent modification in the bird and mammal lineage since the amniote last common ancestor.

Second, episodic memory, of the type where animals answer unexpected questions, has not been reported in amniotes outside of birds and mammals. If episodic memory was in fact present in the amniote common ancestor, and relied on such older amniote structures, we would expect to see reports not only in birds and mammals, but also in other amniotes as well. However, given the fact that episodic memory has only been reported in specific amniotes including birds and mammals, the evidence is more consistent with the idea that the DVR and neocortex each independently implemented a mechanism of episodic memory.

It should also be noted that there are additional tests of episodic memory. For example, one such set of tests of episodic memory has been referred to as tests of “what-where-when” memory, where an animal must remember *what* happened, *where* it happened, and *when* it happened. This type of memory has been challenged as being a true assessment of episodic memory, and it is generally accepted that the “unexpected question” is a better test. “What-where-when” memory has been consistently shown across many phyla, including fish (Hamilton et al., 2016), rats (Bird et al., 2003; Babb and Crystal, 2005; Ergorul and Eichenbaum, 2007), pigeons (Skov-Rackette et al., 2006), apes (Schwartz et al., 2002, 2004, 2005; Mulcahy and Call, 2006), and birds (Zinkivskay et al., 2009) and seems likely to be an ability that emerged far before the first mammals.

#### Possible Adaptive Function(s) of Episodic Memory in Early Mammals (Stem-Group Condition)

It has been proposed that the adaptive benefit of episodic memory is to enable planning about the future (Eichenbaum and Fortin, 2009; Allen and Fortin, 2013; McGaugh, 2013). The ability to remember specific temporal, spatial, and semantic information about events that occurred in a previous situation is useful in anticipating what will happen in a future situation. If planning was a important feature of early mammals, as suggested above, then episodic memory may have been an additional feature that improved planning.

It has also been suggested that episodic memory is useful for remembering social information and hence enables stable social bonds and networks (Emery, 2004; Brennan and Kendrick, 2006; Davidson et al., 2012). The earliest fossil evidence of social behavior is that of early mammals in the Mesozoic (Weaver et al., 2020), suggestive that these early mammals may have been uniquely social compared to earlier amniotes.

#### Conclusion

Taken together, evidence across the in-group, out-group, and stem-group conditions is generally supportive of the hypothesis that episodic memory (of the type where animals answer unexpected questions) emerged within early mammals and evolved independently along the bird line. First, episodic memory has been reported across diverse species of mammals and key neural substrates of episodic memory seem to be structures that emerged within early mammals (in-group condition). Second, the only non-mammal species that have been reported to have such episodic memory are specifically those species known to have independently evolved many unique brain structures and intelligent abilities, such as birds and cephalopods (out-group condition). And third, episodic memory would have offered many adaptive benefits to early mammals, especially if planning head was part of their survival strategy. This hypothesis is consistent with proposals of others (Allen and Fortin, 2013). However, it should be noted that the lack of published evaluations of non-bird reptiles, as well as the lack of studies on the neural substrates of episodic memory in birds, makes this hypothesis tentative.

## Behavioral Abilities That Emerged in Early Primates

**FIGURE 6:**
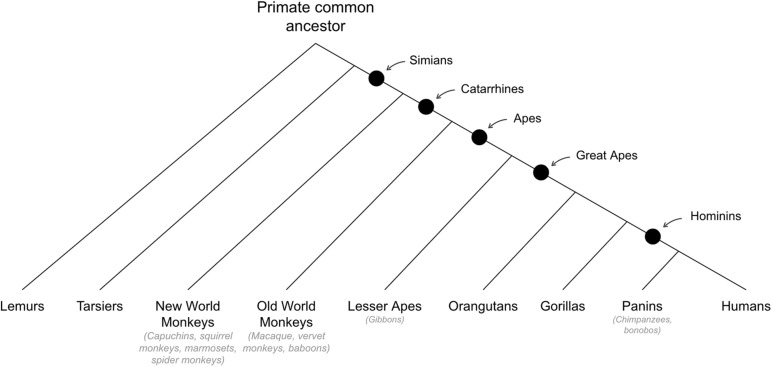
Cladogram of primate divergence.

See Figure 6 for cladogram of primate divergance.

### Hypothesis #9: The Ability to “Anticipate a Need in the Future” Emerged in Early Primates

#### Anticipating Future Needs in Primates (In-Group Condition)

The Bischof–Kohler hypothesis states that humans have a unique ability to make plans to alleviate a need that they will have in the future, but do not currently feel (example: buying food for the week at the grocery store even when not hungry), while other animals are only capable of making plans only to alleviate a need they currently feel (example: generating an optimal path through a maze to get to food when currently hungry as shown in VTE studies) (Bischof-Köhler, 1985). However, the view that only humans can do this has been challenged. Evidence now suggests that many different primates (including chimpanzees, squirrel monkeys, bonobos, and orangutans) are in fact capable of this anticipation of future needs (McKenzie et al., 2004; Mulcahy and Call, 2006; Naqshbandi and Roberts, 2006; Janmaat et al., 2014).

I am not aware of any studies that have examined neural activity during tests of the Bischof-Kohler hypothesis. However, evidence is consistent with the idea that the dorsolateral prefrontal cortex is a substrate of this ability. And crucially, the dorsolateral prefrontal cortex is a structure that emerged in early primates (Semendeferi et al., 2001; Mansouri et al., 2017). The dorsolateral prefrontal cortex of non-human primates and humans seems to activate selectively during situations where you need to take an action to support a need that you will have in the future even if it doesn’t support something you currently want. For example, the dorsolateral prefrontal cortex activates when considering future rewards, but not when considering present rewards (McClure, 2004; Tanaka et al., 2004; Kim et al., 2008). Dorsolateral prefrontal cortex activity is selectively activated when individuals choose delayed rewards over immediate rewards (McClure et al., 2004, 2007; Weber and Huettel, 2008) as well as when successfully avoiding temptation in self-control studies of dieters (Hare et al., 2009). Those with selective inactivation of dorsolateral prefrontal cortex are impaired in their ability to give up immediate rewards for futures ones (Figner et al., 2010). Selective inactivation of dorsolateral prefrontal cortex impairs people’s ability to forgo the excitingly high reward (but high risk) gambles in favor of a lower reward but way lower risk option (i.e., it makes people risk seeking) (Knoch et al., 2006).

#### Anticipating Future Needs in Non-primate Mammals (Out-Group Condition)

I am only aware of one study where the Bischof–Kohler hypothesis was evaluated in non-primate mammals. In this study, non-thirsty squirrel monkeys and rats were tested in their ability to anticipate their future thirst and use this to change their current actions. While squirrel monkeys were shown to successfully anticipate future thirst, rats were incapable of doing so (Naqshbandi and Roberts, 2006). It should also be noted that there is some evidence that birds can solve this task (Roberts, 2007). But as noted above, birds are poor model organisms for the amniote last common ancestor.

#### Possible Adaptive Function(s) of Anticipating Future Needs in Early Primates (Stem-Group Condition)

Early primates are believed to have been foragers who lived in tightly knit social aggregations (Shultz et al., 2011). It has been proposed that living on forest fruits was much more difficult than other forms of foraging (Dunbar and Shultz, 2017). Forest fruits are variable, can face big shortages, are only available and ripe for short periods of time (sometimes only for 72 h, Milton, 1981), and are sought after by many animals (Milton, 1981, 1988; Chapman et al., 1999, 2004; Janmaat et al., 2014). As such, early arrival would be highly adaptive. This may then have required the motivation and ability to get food when it was available even in the absence of hunger (i.e., anticipate a future need).

Consistent with this, it has been shown that larger primate brains help buffer the risks associated with food scarcity during seasonality (van Woerden et al., 2011). Further, chimpanzees have been found to plan their entire foraging path at the beginning of the day to maximize the likelihood of getting food throughout the day (Janmaat et al., 2014). This requires anticipating hunger later in the day even when not yet hungry.

#### Conclusion

Taken together, evidence across the in-group, out-group, and stem-group conditions is generally supportive of the hypothesis that the ability to anticipate future needs is an ability that emerged in early primates. First, the ability to anticipate future needs has been observed in numerous non-human primates and the neural substrates of the ability to anticipate future needs seem to be structure that uniquely emerged in early primates (in-group condition). Second, negative results have been reported in non-primate mammals (out-group condition). And third, there are several plausible proposals as to the unique adaptive benefit of anticipating future needs in the ecological niche of early primates.

### Hypothesis #10: Theory of Mind Emerged in Early Primates

#### Theory of Mind in Primates (In-Group Condition)

Theory of mind refers to the ability of an animal to take the perspective of someone else and understand that they can have different intentions, desires, and knowledge than you do. It continues to be controversial whether any animals other than humans have this ability. But there is compelling evidence that many primates do have theory of mind, even if it is not as robust as in humans. For example, many primates have passed the classic false belief test, whereby animals are tested in their ability to understand that another animal can hold a belief that the individual knows to be false. Macaques (Hayashi et al., 2020), Chimpanzees (Krupenye et al., 2016), and orangutans (Krupenye et al., 2016), have all passed this test.

In another test of theory of mind, non-human primates have been shown to be able to understand the intentions of others, as measured by their ability to distinguish between accidental and intentional actions as well as between someone unwilling to do something and someone unable to do something (Call and Tomasello, 1998; Tomasello et al., 2003, 2005; Call et al., 2004).

Another test of theory of mind is the goggle test, generally regarded to be more difficult than the false belief test. The goggle test includes showing an animal what it is like to look through opaque or transparent goggles, and then seeing if they treat humans wearing these different goggles differently, correctly inferring which human can see through their goggles. Apes have been shown to pass this test (Kano et al., 2019).

Some have proposed that there are degrees of theory of mind, and that while non-human primates have a form of theory of mind, it is far more limited than that of humans. One such interpretation is that non-human primates are aware that others have different beliefs, but only humans after the age of 4 can understand what those false beliefs are and use those beliefs to change their decisions (Kaminski et al., 2008). Although possible, this view is inconsistent with more modern studies that demonstrate the ability of non-human primates to indeed use false beliefs in decisions (Krupenye et al., 2016; Kano et al., 2019), which suggests that non-human primates have more sophisticated theory of mind than previously thought.

Consistent with the view that theory of mind emerged in early primates, the two structures most implicated in theory of mind are the superior temporal sulcus and temporoparietal junction, both of which are structures that emerged within early primates and are not present in non-primate mammals (Kaas, 2009). Theory of mind tasks in humans activates the superior temporal sulcus (Mars et al., 2011, 2012). Superior temporal sulcus activation correlates with subjective reports of considering the point of view of others (Dodell-Feder et al., 2011), when hearing stories (vs. nonsense speech), and observing faces (Beauchamp, 2015). Performance on theory of mind tasks has been shown to correlate with superior temporal sulcus activation (Otsuka et al., 2009). In monkeys, superior temporal sulcus activation is similarly sensitive to social information conveyed by faces, postures, and actions of others (Perrett et al., 1992; Dehaene et al., 2005).

The temporoparietal junction, sometimes considered part of the superior temporal sulcus (Beauchamp, 2015), is also highly implicated in theory of mind tasks. Temporoparietal junction activity in humans is correlated with how likely someone is to give a donation to someone else (Hare et al., 2010; Morishima et al., 2012), how risky a decision is that someone else makes in front of you (van den Bos et al., 2009), as well as altruism in a trust game (van den Bos et al., 2009). It also gets selectively activated when hearing false belief stories (versus false physical stories, Beauchamp, 2015). Although the temporoparietal junction is less studied in non-human primates, connectivity analysis has suggested a temporoparietal junction homolog in monkeys (Mars et al., 2013).

#### Theory of Mind in Non-primate Mammals (Out-Group Condition)

Most studies on non-primate mammals conclude that they do not have theory of mind (Byrne et al., 2001; Tomonaga et al., 2010; Bräuer, 2014; Aldhous, 2015). It should also be noted that there is some evidence of theory of mind in birds (Bugnyar et al., 2016), but the negative results in non-primate mammals, and the evidence of convergent brain evolution in birds, makes this unconvincing evidence for the presence of theory of mind in the amniote common ancestor of birds and primates.

#### Possible Adaptive Function(s) of Theory of Mind in Early Primates (Stem-Group Condition)

Early anthropoids likely lived in social societies with pair-living, had strong family bonds, and foraged in groups (Shultz et al., 2011). Like in the social groups of modern monkeys and apes, these ancestral social groups likely had hierarchies with coalitions and competition. The ability to infer intentions and beliefs of others would have enabled animals to more easily climb social hierarchies, build coalitions, collaborate, and deceive others when necessary (Dunbar and Shultz, 2017). Further, theory of mind may have also been a prerequisite for learning motor skills through observation, which would have been adaptive for group transmission of foraging skills and tool use (see next section).

#### Conclusion

Taken together, evidence across the in-group, out-group, and stem-group conditions is generally supportive of the hypothesis that theory of mind emerged, even in a simple form, with the first primates. First, theory of mind has been observed, even if in a primitive form, within many non-human primates, and the neural substrates of theory of mind in primates seem to be uniquely primate structures (in-group condition), Second, there are numerous negative results of theory of mind in non-primate mammals (out-group condition). And third, there are numerous plausible proposals for the unique adaptive benefit of theory of mind in the ecological niche of early primates.

### Hypothesis #11: Learning Motor Skills Through Observation Emerged in Early Primates

#### Learning Motor Skills Through Observation in Primates (In-Group Condition)

There is evidence that non-human primates can learn motor skills such as tool use and manufacture simply by observing others perform such behaviors. This has been found in macaques (Ferrucci et al., 2019), rhesus monkeys (Meunier et al., 2007), and chimpanzees (Tomasello et al., 1987). For example, young chimpanzees who were able to observe adult chimpanzee use specific tools were able to figure out how to use the tools, while the young chimpanzees who did *not* observe adult chimpanzees use the tools failed to learn how to use them (Tomasello et al., 1987). Further evidence for this learning through observation was confirmed in diffusion experiments, whereby a new technique was taught to a few individuals in a group, and within a short period of time this technique had spread to other individuals in the group (Whiten et al., 2005; Dindo et al., 2009; Gunhold et al., 2014; van de Waal et al., 2015). Transmission of such skills has been shown across generations (Mercader et al., 2007; Haslam et al., 2016; Whiten, 2017).

There is even some emerging evidence that non-human primates will teach skills and tool use to peers and children, although this is still controversial. Such teaching would be a remarkable addition to the learning motor skills through observation ability, whereby one animal can anticipate the motor skills another must learn to achieve a goal. For example, macaques seem to exaggerate flossing when around their young, perhaps to help teach them (Choi, 2009). Chimpanzee’s also show behaviors that some have interpreted as teaching (Everding, 2016; Musgrave et al., 2016). For example, skilled users will bring multiple fishing probes to a fishing activity and give one to a youngster. They will divide one in two if the child does not have one and will respond to begging if the child needs a tool. They can even identify that a youngster is struggling and will swap tools with them. But such teaching in non-human primates has been disputed (Premack, 2007; Hoppitt et al., 2008; Kline, 2015).

Further evidence for the idea that learning motor skills through observation emerged in early primates can be seen in the observation that many neural substrates of learning through observation are uniquely primate structures. The superior temporal sulcus in both humans and non-human primates activates in response to observing various forms of biological motion (Perrett et al., 1985; Puce and Perrett, 2003). Further, individual superior temporal sulcus neurons are highly selective for specific actions, as opposed to just being activated generically whenever biological movement is occurring (Perrett et al., 1985; Puce and Perrett, 2003). Superior temporal sulcus neurons are selective to the observation of others, and don’t respond when the individual does the same action that they observed (Perrett et al., 1989; Jellema et al., 2000).

Humans and non-human primates have also been shown to also have mirror neurons in the premotor cortex and parietal lobes that activate both when an individual is taking a specific action as well as when they observe other conspecifics doing that same action (Gallese et al., 1996; Rizzolatti et al., 1996; Fogassi, 2005). Observed motor acts in others done by different body parts are somatotopically organized in the classic homunculus the same way as when the individual moves those same body parts themselves (Penfield and Rasmussen, 1950; Woolsey et al., 1952; Buccino et al., 2001). Interestingly, these mirror neurons respond robustly even if non-human primates can only infer the motion of others without explicitly observing it, such as when half of the motion is obscured (Umiltà et al., 2001; Kohler et al., 2002). The general view is that these mirror neurons encode the abstract goals of motor acts (Rizzolatti et al., 2001). Recording studies in non-human primates corroborate this view, as mirror neurons seem to discriminate different goals, even if the observed movements are the same (Fogassi, 2005).

As suggested by others (Tennie et al., 2009; Tomasello and Moll, 2009; Dean et al., 2013), it is likely that these abilities in non-human primate is not as sophisticated as they are in humans. One difference that has been proposed is that of shared intentionality, whereby although non-human primates understand intentions of others, they are much less motivated than are humans to share their mental states (Tomasello and Carpenter, 2007; Call, 2009). Another difference that has been suggested is that observational learning may be much more cumulative in humans than in non-human primates (Tennie et al., 2009; Dean et al., 2013). For example, although chimps can learn through observation, and can transmit these across transmission chains (Whiten et al., 2005), it has been suggested that such learning tends to be focused on the outcomes of movements, as opposed to also mimicking the entire nuanced movements themselves. This prevents a cumulative evolution of an action or cultural phenomena, since it always falls back to individuals finding the easiest way to achieve an outcome. Individuals will happily modify a learned skill to make it easier for themselves. It has been suggested that in humans however, there is a cumulative mimicry of actual actions, independent of outcome, which then creates a unique “ratcheting up the rachet” (Tennie et al., 2009) of culture, motor skills, and information. In contrast, in non-human primates such knowledge, while still transferable across generations, does not accumulate across generations as accurately.

#### Learning Motor Skills Through Observation in Non-Primate Mammals (Out-Group Condition)

Many animals, even fish, demonstrate socially coordinated movements. Coordinating movements with other conspecifics is likely a key anti-predator behavior evolved very early in evolution, perhaps even in early vertebrates. Further, there is also evidence of learning paths by observation in early diverging vertebrates such as fish (Lindeyer and Reader, 2010; Brown, 2015) and reptiles (Wilkinson et al., 2010), where they will learn to take navigational paths through simply observing conspecifics take the same path.

However, there are two differences between observational learning in non-human primates relative to that in non-primate vertebrates. First, learning through observation of the type in non-human primates includes an understanding of the intention of movement, not simply mirroring the movement. Coordination and path following on the other hand can be achieved with much simpler mimicry. I am not aware of any studies demonstrating the ability to infer intentions in non-primate mammals. Further, goal-selective mirror neurons found in primates have not been found in other non-primate mammals. Although mirror neurons have indeed been found in songbirds (Prather et al., 2008), but as discussed previously, birds are a poor model organism for inferring abilities in our amniote common ancestor. Second, learning through observation in non-human primates has been shown to be incredibly transferable, and can be transmitted not only amongst peers but across many generations. Fish that learn paths through observation do not pass down this knowledge from generation to generation (Lindeyer and Reader, 2010).

#### Possible Adaptive Function(s) of Learning Motor Skills Through Observation in Early Primates (Stem-Group Condition)

As observed in many extant primates, it is likely that early primates (or at least anthropoids) used sophisticated motor skills and tools to obtain food from hard-to-get-places that other animals can’t access. It is then reasonable to speculate that the ability for such skills to be transmitted throughout social groups would have improved a groups’ ability to reliably obtain food.

#### Conclusion

Taken together, evidence across the in-group, out-group, and stem-group conditions is generally supportive of the hypothesis that learning motor skills through observation emerged in early primates. First, there are numerous reports of learning through observation throughout non-human primates, and the neural substrates of learning through observation seem to be structures that emerged first in early primates (in-group condition). Second, the observational learning in non-primate mammals seems to be much more limited (out-group condition). Specifically, non-primate mammals seem to lack the ability to understand the intention of observed motor movements as well as to consistently transfer such skills across generations – two features of observational learning consistently seen in non-human primates. And third, learning motor skills through observation would have offered adaptive benefits to the foraging ecological niche of early primates.

## Behavioral Abilities That Emerged in Early Humans

### Hypothesis #12: Language Emerged in Early Humans

#### Language in Humans (In-Group Condition)

The ability to name objects and organize words with grammar has been suggested to be what makes human language unique (Berwick and Chomsky, 2017; Terrace, 2019). The ontogeny of human language learning is revealing as to how these unique features of language emerge. Human infants even as young as a few months, far before they can speak, engage in affective imitation and rhythmic exchanges with their mother of various affective forms of communication – such as vocalizing, making gestures and facial expressions (Meltzoff and Moore, 1977, 1989). Human infants and mothers will match the duration of each other’s pauses, creating affective proto conversations (Beebe et al., 1988, 2016). By 6 months of age, infants begin to engage in *shared attention* of the same object as their mother. Infants have non-verbal mechanisms to confirm that they saw what you saw (Carpenter and Call, 2013). This enables parents to name things that have joint attention. As evidence for its role in language, the more joint attention expressed by a child, the larger the child’s vocabulary 12 months later (Morales et al., 2000; Mundy et al., 2007). This is suggestive that two abilities required for the development of language are rhythmic exchanges of affective communication (proto conversations) and shared attention.

The two areas most implicated in language are Broca’s area in on the left inferior prefrontal cortex and Wernicke’s area in the left temporal lobe. Lesions to both these areas severely impair language abilities (Wernicke, 1995; DeWitt and Rauschecker, 2013). However, these areas have homologous regions in non-human primates, making it unlikely that these are completely new structures from which language emerged. It is more likely that language emerged via new connectivity that used existing structures in new ways.

The most notable connectivity difference is seen in the arcuate fasciculus, which connects Broca’s area and Wernicke’s area. In humans the arcuate fasciculus is massively expanded (Aboitiz and García, 1997; Aboitiz et al., 2006, 2010; Rilling et al., 2008, 2012; Aboitiz, 2012; Petrides, 2014; Rilling, 2014; Catani and Bambini, 2014; Stout and Hecht, 2017). The arcuate fasciculus also appears to contain unique connectivity in humans – whereby the frontal cortex of left hemisphere connects to left medial temporal gyrus and inferotemporal gyrus, close to the areas usually included in Wernicke’s area – a connection not observed in non-human primates (Rilling et al., 2008). The proper functioning of arcuate fasciculus is also highly associated with various functions of language (Binder and Desai, 2011; Schomers et al., 2017). Damage to arcuate fasciculus impairs verbal working memory, fluency, and comprehension. Further, the development of AF correlates with language abilities in childhood (Friederici, 2011; Yeatman et al., 2011; Skeide et al., 2016; Goucha et al., 2017; Schomers et al., 2017), and the strength of connectivity of left arcuate fasciculus correlates with performance in word learning (Lopez-Barroso et al., 2013).

#### Language in Non-human Primates (Out-Group Condition)

Many animals communicate with one another – birds sing songs to attract mates and defend territories (Langmore, 1998), bees communicate location using dances (Aguilar et al., 2005), and vervet monkeys have alarm calls specific to different predators (Cheney and Seyfarth, 1996). But human language differs from these forms of communication in that humans have grammar and words. Human language can combine sounds (phonemes and words) using grammar to create an almost infinite number of novel meanings. The results from attempts to teach non-human primates such flexible language have mostly supported the conclusion that non-human primates are incapable of learning language with grammar and words. Initial potential positive results in teaching the basics of sign language (Gardner and Gardner, 1969; Patterson, 1978) have been disconfirmed by later studies (Terrace et al., 1979), where it was shown that signing was always a sequence of prompted signs, had low diversity, and was non-grammatical. Further analysis of these earlier studies also demonstrated that the subjects only learned sequences of symbols to get rewards, and sequences were the result of simple non-grammatical rules (Thompson and Church, 1980). Some of these earlier studies may even have unintentionally cued the animals to make specific signs (Miles, 1983). Later work with bonobos showed impressive signing abilities, perhaps indicative of simple grammar and words (Savage-Rumbaugh et al., 1986), but this interpretation is still controversial (Pinker, 1994; Terrace, 2019). The most popular view seems to be that non-human primates can learn imperative functions of symbols (use them to attain rewards), but they fail to learn that objects have names, and that those names can be used conversationally (Terrace, 2019).

The underlying ontogenetic mechanisms whereby language emerges in infant humans, namely joint attention and proto-conversations, are not present in non-human primates. Attempts to show joint attention in chimps showed negative results (Warneken, 2006; Warneken et al., 2006).

There are no reports of these proto-conversations in non-human primates and others have suggested they do not occur (Terrace, 2019). It has been shown that collaborative activities that require joint attention become much more difficult for non-human primates than those that don’t require joint attention (Tomasello and Carpenter, 2005; Tomasello et al., 2005; Melis and Tomasello, 2013).

Further evidence for the uniqueness of human language relative to animal communication can be seen in their completely different neural substrates. Primate calls, such as those in vervet monkeys, are driven by midbrain and limbic forebrain structures (Jürgens, 1988). Damage to monkey cortical areas homologous to human language areas do not produce call production deficits (Aitken, 1981; Jürgens et al., 1982). And voluntary speech in humans is driven by direct cortical projections. Circumventing the limbic and midbrain pathways wherein animal calls tend to be produced (Fitch, 2018; Jarvis, 2019). This is strong evidence that language is not an evolutionary elaboration of animal call production system, but a separate system all together.

#### Possible Adaptive Function(s) of Language in Early Humans (Stem-Group Condition)

There are numerous proposals for the original adaptive benefit that language provided early humans, including cooperative hunting (Washburn and Lancaster, 1968), promotion of pair bonding (Deacon, 1997), expediting of toolmaking (Greenfield, 1991), and the enhancement of teaching (Laland, 2017). One additional leading theory is the confrontational scavenging theory, which suggests that the ecological niche of early humans was to split up into groups to scout for dead animals. After finding a carcass they would recruit a large group to scare off other animals and work together to scavenge the remains. This would have required *words* to reference the identity and location of the discovered remains to other conspecifics (Terrace, 2019), this ability has been called displaced reference, whereby an animal can refer to something that is currently not present (Blumenschine et al., 1994). Note that select invertebrates seem to have independently evolved certain abilities of displaced reference as well, although it seems reflexive and not flexible as in humans, including bees (Von Frisch, 1967) and ants (Wilson, 1962).

#### Conclusion

Taken together, evidence across the in-group, out-group, and stem-group conditions is generally supportive of the hypothesis that the ability to use language with words and grammar first emerged in early humans. First, language seems to be universal across human cultures, and key neural substrates of language in humans seem to use modifications that emerged uniquely in early humans (in-group condition). Second, most studies of language in non-human primates conclude that they are unable to learn language with words and grammar (out-group condition). And third, evidence suggests that the ecological niche of early humans was such that language would have offered a uniquely adaptive benefit (stem-group condition). Additionally, the ontogeny of language in humans seems to rely on proto-conversations and joint attention, two features that have been reported to not exist in non-human primates.

### Hypothesis #13: Music Emerged in Early Humans

#### Music in Humans (In-Group Condition)

Music, like language, seems to be universal to humans – music is found across all human cultures, and across all these cultures music shares many features (Brown and Jordania, 2011). One feature of music across human cultures is beat-based timing. Evidence suggests that beat-based timing has a separate neural substrate than duration-based timing (Teki et al., 2011). Duration-based timing requires discriminating the absolute amount of time passed, whereas beat-based timing requires discriminating relative timing. Duration-based timing seems to be implemented in the inferior olivary nucleus and cerebellum (Teki et al., 2011). In contrast, beat-based timing seems to be implemented within a network of the striatum, thalamus, premotor cortex, and prefrontal cortex (Rao et al., 1997; Jäncke et al., 2000; Grahn and Brett, 2007; Wiener et al., 2010; Teki et al., 2011). The mere perception of a beat, even without movement, activates premotor areas (Grahn and Brett, 2007; Chen et al., 2008; Grahn and Rowe, 2009; Geiser et al., 2012; Teki et al., 2012; Kung et al., 2013) and creates enhanced coupling between auditory areas and motor areas (Kung et al., 2013). In humans this circuitry is involved in many observed beat-related behaviors such as dance, music, timing movements, and sequencing movements (Grafton et al., 1995; Grahn and Brett, 2007; Harrington et al., 2009; Wiener et al., 2010). Consistent with this, humans with cerebellar lesions are impaired in duration-based timing tasks, but not in beat-based timing tasks (Grube et al., 2010). And lesions of striatum or substantia nigra impair beat-based timing (Teki et al., 2011). Further, Parkinson’s patients, who have impairment in basal ganglia function, also seem to struggle with beat-based timing tasks (Teki et al., 2011).

#### Music in Non-human Primates (Out-Group Condition)

All mammals, especially non-human primates, have very impressive auditory perception. They can perceive pitches (Song et al., 2015) and they can learn very subtle differences in sound structures (Selezneva et al., 2006; Tsunada et al., 2011). Many mammals and birds can perceive the fundamental frequency of a chord even if it has been removed (showing complex pitch perception) (Heffner and Whitfield, 1976; Cynx and Shapiro, 1986; Tomlinson and Schwarz, 1988). Non-human primates can also readily observe the interval timing between two events, such as two clicks, and repeat back almost the same interval (Zarco et al., 2009).

However, there are differences in music-related cognitive abilities between humans and non-human primates. The most obvious difference is with beat perception. Monkeys cannot learn to synchronize taps with an auditory or visual metronome, even after a year of training (Zarco et al., 2009; Honing et al., 2012). Electro-encephalogram studies in non-human primates have confirmed that they do not seem to perceive beats in auditory signals (Honing et al., 2012). Even birds, bats, and dolphins with exceptional natural song abilities, have weak beat perception (Patel et al., 2009; Schachner et al., 2009; Merchant and Honing, 2014). Moreover, this very basic skill of tapping to a beat is remarkably universal and accurate amongst human (Wallin et al., 2000; Iversen and Patel, 2008; Rankin et al., 2009; Repp and Su, 2013; van der Steen and Keller, 2013). Human beat perception is also flexible: humans can adjust the same melody to changing beats across a very wide range of tempos (Large and Jones, 1999; Honing, 2013). Even the few animals that show some form of rhythmic entrainment (such as birds), do not show the ability to synchronize across a wide range of tempos as humans do (Large, 2000; Fitch, 2009; Patel et al., 2009).

The cortico-basal-ganglia-thalamic circuit important for human musical ability also exists in monkeys and is also related to timing and sequencing movements (Tanji, 2001; Merchant et al., 2013; Perez et al., 2013). However, there are differences in the circuitry between humans and non-human primates that may explain the lack of beat-perception in non-human primates. First, the arcuate fasciculus in humans connects auditory superior temporal areas and premotor areas much more strongly than in non-human primates. Broca’s area (as well as homolog on right side), known to be less developed in non-human primates (Petrides and Pandya, 2009), has been implicated in concatenating and organizing hierarchical temporal sequences of simple motor actions (Koechlin and Jubault, 2006). Second, superior temporal areas in humans project much more extensively to the basal ganglia than they do in macaques (Yeterian and Pandya, 1998; Borgmann and Jürgens, 1999; Rilling et al., 2008; Thiebaut de Schotten et al., 2012). Consistent with this, the cortico-basal-ganglia-thalamic circuit is activated less by audiomotor tasks in Rhesus monkeys than in humans (Grahn and Brett, 2007).

Non-human primates also struggle with *relative* pitch perception more than humans do. Non-human animals that learn melodies often struggle to generalize these to transpositions (Hulse et al., 1984; D’Amato, 1988), a task that is mostly automatic for humans. The reports that demonstrate basic forms of relative pitch perception (Ferrets: Yin et al., 2010; Rhesus monkeys: Wright et al., 2000; Dolphins: Ralston and Herman, 1995) use very simple paradigms, where a subject merely as to perceive an octave generalization or to simply discriminate increasing pitch versus decreasing pitch– and even learning these simple tasks often requires a lot of training. Further, evidence suggests non-human primates show no preference for consonance, a feature of certain pitch intervals that humans across cultures tend to report finding pleasant (McDermott and Hauser, 2004; Vassilakis, 2005). Although some birds do show this preference (Chiandetti and Vallortigara, 2011).

#### Possible Adaptive Function(s) of Music in Early Humans (Stem-Group Condition)

Numerous adaptive benefits have been proposed for music, most of which hinge on the social interactions of early humans (reviewed in Cross and Morley, 2008). For example, it has been proposed that music was useful for coordinating cooperative social behaviors (Brown, 2006; Cross, 2009), for more nuanced forms of communication that language alone did not sufficiently satisfy (Cross, 2009), or for promoting group cohesion (Roederer, 1984). Musical instruments have been found over 50,000 years ago, suggesting that music emerged around a similar period as language (Cross, 1999; Morley, 2003).

#### Conclusion

Taken together, evidence across the in-group, out-group, and stem-group conditions is supportive of the hypothesis that the totality of human-level music perception, inclusive of beat perception, relative pitch, and consonance preference, emerged in early humans and did not exist in our last common ancestor with chimpanzees. First, music has been observed across human cultures, suggesting that it is universal to humans and key neural substrates of music include neural modifications that are unique to humans (in-group condition). Second, negative results of various features of music, such as beat perception, have been reported in non-human primates (out-group condition). And third, the ecological niche of early humans was such that music may have offered unique adaptive benefits (stem-group condition).

## Discussion

I have presented 13 hypotheses of specific behavioral abilities that emerged at major milestones in the evolution of the human brain. I hope they provide a guide for further comparative analysis so they may be refined as more evidence emerges. Deciphering the phylogenetic history of human intelligence offers many opportunities to better understand both our own history and how the human brain works.

### Comparison to Other Work

Ginsburg and Jablonka (2019, 2021) have done extensive work in chronicling the evolutionary transitions by which various learning abilities emerged. The hypotheses presented here were informed by their work. I have attempted to add to their framework by adding specific behavioral capabilities that likely emerged at each transition, in addition to various features of learning. They define three levels of learning: non-associative learning (sensitization and habituation), limited associative learning (LAL), and unlimited associative learning (UAL). They describe LAL as the ability to reinforce the association between “simple” stimuli and actions. This is what I have called “associative learning” in hypothesis #2. They define UAL as a form of learning that enabled *unlimited* numbers of associations between complex stimuli and actions. They argue that UAL enabled four key advantages: (1) animals could perceive compound stimuli and actions, (2) animals could engage in pattern completion, (3) animals could make guesses as to the right behavior based on past experiences, and (4) animals could make cumulative improvements to cope with new situations. They argue that UAL independently evolved in three lineages. The hypotheses here parse out some of the specific features of UAL that emerged within the vertebrate lineage – such as omission learning, map-based navigation, VTE, and episodic memory. Further, some abilities presented here are not “learning” abilities *per se*, and hence are additive to their analysis. For example, the ability to anticipate a need in the future or theory of mind are not necessarily only unique in the ability to *learn* something new, but also the ability to *apply* something previously learned in a new way.

Cisek’s (2007, 2019) work on phylogenetic refinement also hypothesizes specific initial behaviors that were present in ancestral species. Some of the hypotheses here are consistent with his. For example, his proposal that early bilaterians had mechanisms for exploitation and exploration, modulated by neuromodulators and peptides that signaled internal states, is consistent with the hypotheses here that taxis navigation with valence and cross-modal integration first emerged in early bilaterians. Cisek also presents various interesting ideas on the sensorimotor and other functions of the neocortex, which are not explored in this paper, such as his theory of affordance competition, whereby the neocortex provided novel mechanisms for flexible action specification and selection (Cisek, 2007; Cisek and Kalaska, 2010; Pezzulo and Cisek, 2016).

There has also been work done chronicling the evolution of memory systems (Murray et al., 2017). Relevant hypotheses here are largely consistent with their arguments. For example, they similarly propose that a spatial navigation system emerged in early vertebrates. Further, they similarly propose that granular prefrontal areas that emerged in early primates enabled more complex goal representations, consistent with the idea that granular prefrontal areas were supportive of anticipating future needs. They also propose unique features that emerged in the primate lineage (not discussed in the current paper), such as grasping and manual foraging mechanisms. The hypotheses here provide additional proposals for behavioral abilities that emerged.

### Testable Predictions

Testable predictions can be formulated for each of the hypotheses presented here. The three conditions (in-group, out-group, and stem-group conditions) provides a framework for formulating such predictions; each condition has a corresponding set of predictions. Consider the hypothesis that omission learning emerged in early vertebrates. Testable predictions of the in-group condition are that additional early diverging vertebrates will succeed in omission learning studies. Testable predictions of the out-group condition are that additional tests of protostomes will demonstrate failures in omission learning, or if they succeed, it will be implemented in neural substrates that independently evolved. Testable predictions of the stem-group condition are less specific but can be formulated as the prediction that the ecological niche of early vertebrates will prove to have been consistent with an environment where omission learning would have been adaptive.

## Data Availability Statement

The original contributions presented in the study are included in the article/supplementary material, further inquiries can be directed to the corresponding author/s.

## Author Contributions

MB conceived the overall theory and wrote the entire manuscript.

## Conflict of Interest

The author declares that the research was conducted in the absence of any commercial or financial relationships that could be construed as a potential conflict of interest.

## Publisher’s Note

All claims expressed in this article are solely those of the authors and do not necessarily represent those of their affiliated organizations, or those of the publisher, the editors and the reviewers. Any product that may be evaluated in this article, or claim that may be made by its manufacturer, is not guaranteed or endorsed by the publisher.
